# Perturbation responses in co‐evolved model meta‐communities

**DOI:** 10.1002/ece3.9534

**Published:** 2022-11-21

**Authors:** Gavin M. Abernethy

**Affiliations:** ^1^ Computing Science and Mathematics University of Stirling UK

**Keywords:** invasion, meta‐community model, patch removal, perturbation, reserves

## Abstract

A spatially explicit eco‐evolutionary model assembles simulated meta‐communities which are subjected to species and community perturbation experiments to determine factors affecting the stability of the global ecosystem. Spatial structure and resource variety increase the persistence of the ensembles against the removal of an individual species, yet they remain vulnerable to re‐invasion by an existing member of the meta‐community if it is introduced to all patches with minimal population. Optimal reserve placement strategies are identified for maximally preserving global biodiversity from the effects of sequences of patch disruption, and targeted reserve placement that shields the most or the rarest biodiversity is usually effective. However, if disturbed populations are permitted to re‐settle in neighboring patches, then reserves should also be situated remotely to isolate their residents from invasion.

## Graphical abstract

A spatially‐explicit eco‐evolutionary model assembles a set of meta‐communities subjected to species and patch‐level perturbations. Optimal reserve placement strategies are identified for maximally preserving global biodiversity from sequential patch disruption.

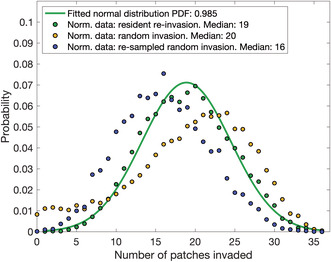

## 1 BACKGROUND

Anthropogenic impact on the natural environment is a pressing concern for the preservation of global biodiversity (Pimm & Raven, 2000), resulting in the need to understand how ecosystems will respond to the loss or invasion of species and the fragmentation or removal of habitats in order to manage sustainable land use. Given the practical and ethical obstacles to direct experimentation, predicting the impact of perturbations may be undertaken using mathematical models of community ecology (Keddy, 1992), but there remains a need for improved understanding of the mechanisms at work in such complex systems (Montoya, 2021). The use of models to inform population management is well established in fisheries, illuminating how over‐harvesting impacts the size structure of marine communities (Fung et al., 2013) and how species loss corresponds to the degradation of ecosystem services (Keyes et al., 2021). For terrestrial ecosystems, strategic placement of nature reserves will be essential to maximally preserve threatened species (Venter et al., 2014), with a preference for one large or many small reserves dependent on whether migration between reserve fragments is possible (Pelletier, 2000). Conservation studies may use GIS data to identify optimal wildlife corridors for mammals (Penjor et al., 2021) in forests (Yemshanov et al., 2021) and urban ecological networks (Zhao et al., 2019).

To simulate habitat degradation, fragmentation, or removal it is necessary to represent spatial structure and processes within ecological models (Howell et al., 2018). Landscape ecology (Erös & Lowe, 2019) utilizes detailed and continuous models of the physical landscape. Alternatively, meta‐population models divide the environment into discrete habitat units where sub‐populations operate, facilitating the study of colonization–extinction dynamics. Single‐species models are frequently used to study the role of dispersal rates, environmental heterogeneity (McManus et al., 2021), and trade‐offs between specialist and generalist strategies (Szép et al., 2021). These techniques can further extend community models to meta‐community models of many species interacting in a spatially explicit environment (Gravel et al., 2016; Gross et al., 2020; Osakpolor et al., 2021; Pillai et al., 2010), with such models demonstrating how environmental heterogeneity can promote biodiversity (Ryser et al., 2021). Dispersal and meta‐community dynamics alone are sufficient to account for complex food web networks (Pillai et al., 2011). The topology of the spatial network mediates the relative roles of mass effects and species sorting in spatial pattern formation (Suzuki & Economo, 2021), and the successfulness and impact of species invasions (Häussler et al., 2021) to govern the structure of linked communities (Gross et al., 2020). In particular, functionally large distances separating species in space facilitates co‐existence for less‐competitive generalists and thus enables greater biodiversity (Barter & Gross, 2017).

Food web community structure is known to impact the response of model communities to habitat loss (Melián & Bascompte, 2002), while spatial structure influences the response of each community in a network (Gross et al., 2020) to disturbance with mechanisms such as rescue effects and source–sink dynamics that are not possible without explicit spatial modeling. Furthermore, it is increasingly recognized that the non‐random network structures of real food webs enhance their stability (Martinez et al., 2006; Yodzis, 1981), and hence model communities should be ecologically plausible in order to observe a realistic response to perturbation. Previous studies have used population dynamics on food webs specified by hand (Eklöf & Ebenman, 2006) or constructed with assembly models (Berg et al., 2015). However, recent work has extended eco‐evolutionary community models with the explicit spatial structure to develop a class of eco‐evolutionary meta‐community models (Abernethy, 2021; Abernethy et al., 2019b; Allhoff, Weiel, et al., 2015; Bolchoun et al., 2017; Hagen et al., 2021; Hamm & Drossel, 2021; Rogge et al., 2018). While not readily amenable to mathematical analysis, co‐evolved model ecosystems display more realistic structural patterns emerging from their evolutionary dynamics (Allhoff, Ritterskamp, et al., 2015; Loeuille & Loreau, 2005, 2009) than can be achieved by models of community assembly via invasions from a pre‐defined pool (Romanuk et al., 2019). They may embed complex relationships and dependencies that would not be obvious to identify and encode, and which may influence their response to disturbance by improving stability (Emary & Evans, 2021). For example, the crucial stabilizing role of weak feeding links has been identified for both real (Neutel et al., 2002) and model (McCann et al., 1998) food webs. This combination of meta‐community dynamics and assembly rules can successfully generate observed macroecological patterns of species distributions (O'Sullivan et al., 2019). Consequently, future model investigations of ecosystem responses to perturbation, and especially to habitat loss, should include spatial processes and realistic community structure that may be obtained by an explicit modeling of evolutionary processes.

Ecosystem perturbation includes the possibility of the unexpected extinction of a species, invasion by an exotic species, or the degradation of a habitat or its removal altogether. The impact of removing one or a sequence of species from a community has been well‐studied in model ecosystems, beginning with the sequential deletion of species from purely topological representations of empirical food webs (Dunne et al., 2002; Keyes et al., 2021). The deletion of a single species has been studied in hand‐made models with simple Lotka–Volterra population dynamics (Eklöf & Ebenman, 2006), and in some eco‐evolutionary model communities (Quince et al., 2005). Until 2015, few studies had simulated sequences of species loss from models incorporating population dynamics (Berg et al., 2015; Jonsson et al., 2015), and these revealed that including relatively simple dynamics can yield different stability outcomes to the previous investigations. Similarly, it may be that including more realistic population dynamics, co‐evolved community structure, and explicit spatial effects may demonstrate new outcomes again, indicating the need to expand similar perturbation experiments to models of complex co‐evolved meta‐communities. Habitat loss perturbations may be in the form of patch removal, or patch fragmentation where connections between occupied sites are broken. This is a known concern for biodiversity management (Haddad et al., 2015), with many empirical studies reported (Debinski & Holt, 2000). These have demonstrated the need to preserve the spatial size of existing habitats as smaller stream habitats have reduced stability to perturbation (Greig et al., 2022), while the severity of the perturbation influences the subsequent recovery of termite and soil communities (Davies et al., 1999). To complement these, simulation models have been developed to investigate the impact of habitat loss on target species (Michael Reed et al., 2002), and have successfully generated qualitative predictions of the impact of habitat loss—for example, on white‐footed mice (Burns & Grear, 2008), provided that accurate assumptions about species re‐settlement behavior are incorporated. However, studies of habitat loss on model meta‐communities usually feature only a few (2–4) species in pre‐defined relationships (Bascompte & Solé, 1998; Liao et al., 2017; Melián & Bascompte, 2002; Moilanen & Hanski, 1995; Nee & May, 1992), or use alternative individual‐based approaches (Furness et al., 2021). A practical application of spatial stability modeling for conservation and management is the question of optimal placement of reserves, but reserve decision models typically lack the multi‐species population dynamics that may influence optimal site selection (Williams et al., 2005). Recent patch fragmentation models have started to incorporate larger statistically generated communities (Ryser et al., 2019), but so far habitat loss has had little attention in eco‐evolutionary meta‐community models and none have considered reserve placement.

This study uses a previously described spatial eco‐evolutionary model (Abernethy, 2020) to generate a set of abstract trait‐based meta‐communities which are subjected to perturbations at both the species and patch level and tested for optimal placement of reserves in the spatial network. This has been developed from an extension of the Webworld eco‐evolutionary model (Drossel et al., 2001). As such, it incorporates the feedback of ecological and evolutionary processes to generate ecologically plausible food web structures, and the extension to a 36‐patch spatial lattice allows for explicit spatial processes and the direct testing of habitat perturbations. In addition to the applied ecology and conservation literature, the response of model communities to perturbation is also a question that arises out of the stability–complexity debate (McCann, 2000) in the study of complex networks (ecological and otherwise), and this model has its heritage in the statistical physics literature. The response of its generated communities to species deletion (Quince et al., 2005) and patch disturbance (Abernethy, 2021) has been previously investigated, and this work will further generalize how such eco‐evolutionary models respond to perturbation while also addressing the need described above for more developed modeling solutions to the questions of applied ecology. Here, we improve the species range by a modified dispersal function and investigate the role of species properties in invasion dynamics, how the distribution of species governs the relative impact of different types of patch‐level disturbance, and the optimal placement and connectedness of nature reserves in the spatial meta‐network. In §2, the model is described in full, and §3 describes the pre‐perturbation properties of the assembled meta‐communities. Perturbations at the species level are studied in §4–5, considering the impact of species removal or invasion. Patch‐level disruptions are investigated in §6–7, where we study the impact of removing single or sequences of multiple patches, and the optimal selection of patches as nature reserves that cannot be directly perturbed.

## 2 MODEL DESCRIPTION

The spatial eco‐evolutionary model used to generate the trophic meta‐communities is a modification of that employed previously in Abernethy (2021) based on the original formulation by Drossel et al. (2001). The model design was previously explained in (Abernethy, 2020), with the main distinction being the formulation of the rules governing the movement. We provide a summary sufficient for implementation here.

Each independent simulation generates a meta‐community of species arranged in 36 coupled communities from a single initial species and 36 resources—one each upon a two‐dimensional 6 × 6 spatial grid of patches. Each species is defined by the 10 discrete traits that they possess from a pool of 500, and a single continuous bodysize. At the initialization of the simulation, an antisymmetric matrix *β* of scores between 0 and 1 is generated, and 10 traits are drawn for each resource species (with default bodysize of 1) and for the initial species which has bodysize $s_{1}=s_{0}=\exp\left(1\right)$ and initial population of $N_{1,1,1}=1$ in patch $\left(1,1\right)$. As each patch resource is generated independently and randomly, the environment is heterogeneous with low spatial autocorrelation. The simulation consists of a series of nested loops representing ecological and evolutionary processes operating on different time scales. On the outermost layer, the meta‐community of species is assembled from the initial species over 10,000 evolutionary time steps. Due to the process of three nested loops, the process is very computationally expensive when there are hundreds of sub‐populations of species interacting within many local communities, so the restriction to 15 assembled meta‐communities is a practical limitation. However, it results in 540 local communities that have co‐evolved over many speciation events and dispersal processes and consist of 7022 species divided into over 11,000 local sub‐populations. Furthermore, 10,000 evolutionary time steps are significant compared to previous implementations of this class of models, and the model has been demonstrated to generate a constant turnover of species compositions (Drossel et al., 2001) failing to reach an equilibrium even after $10^{8}$ speciations (Abernethy et al., 2019a).

### 2.1 Evolutionary time step

Each evolutionary time step consists of 1000 ecological time steps, followed by a speciation event. Here, the local population of one non‐resource species is randomly selected to be the parent species with probability in proportion to the population size, and a value of 1 is deducted from its population. A child species with a population of 1 is introduced to that patch, inheriting nine traits from its parent species while a randomly selected trait is replaced by a different random choice from the pool of 500. The bodysize of the child is uniformly drawn from an interval $\left[0.8,1.2\right]$ multiplied by the bodysize of the parent. New feeding and competition scores are then calculated for the new child species, and the ecological rules of the system dynamically determine whether or not this species is able to establish itself in the local community, and the emergent consequences on the existing network and meta‐network of species.

### 2.2 Ecological time step

An ecological time step consists of 1000 foraging time steps, where the local populations of all species i in all patches simultaneously decide on their feeding strategies. When this is completed, the population $N_{i,x,y}^{t}$ of species i in the patch $\left(x,y\right)$ at ecological time step t are updated according to the allometric population dynamics:

(1) $$N_{i,x,y}^{t}\mapsto N_{i,x,y}^{t}+\Delta \left(-2s_{i}^{-0.25}N_{i,x,y}^{t}+\lambda s_{i}^{-1}N_{i,x,y}^{t}\sum_{j=0}^{n}g_{i,j}s_{j}-\sum_{k=1}^{n}N_{k,x,y}^{t}g_{k,i}\right)$$

where λ=0.3 is the ecological efficiency (the proportion of consumed biomass available for conversion to predator biomass), an increase relative to non‐allometric model variants in order to recover realistic food web structure, as turns out to be necessary for other designs of allometric eco‐evolutionary meta‐foodweb models (Hamm & Drossel, 2021). Δ=0.1 is the Euler timestep. Within the brackets of Equation (1), the three terms describe the change in population due to natural mortality, population gain due to feeding on the resource or other species, and population loss due to predation from other species, respectively.

Subsequently, dispersal to neighboring patches may occur and the local populations are updated again:

(2) $$N_{i,x,y}^{t}\mapsto N_{i,x,y}^{t}+\sum_{j=1}^{x_{\max}}\sum_{k=1}^{y_{\max}}\delta_{j,k,x,y}\mu_{i,j,k,x,y}N_{i,j,k}^{t}-\sum_{j=1}^{x_{\max}}\sum_{k=1}^{y_{\max}}\delta_{x,y,j,k}\mu_{i,x,y,j,k}N_{i,x,y}^{t}$$

In Equation (2), the two double‐summation terms encode immigration to and emigration from the patch $\left(x,y\right)$, respectively, where $\mu_{i,j,k,x,y}$ is the fraction of the local population of species i in patch $\left(j,k\right)$ that moves to the patch $\left(x,y\right)$. Populations may only move between patches that are directly adjacent on the two‐dimensional spatial lattice, and are prohibited from moving diagonally, so $\delta_{j,k,x,y}=1$ if and only if either ∣j−x∣=1 and k=y or j=x and ∣k−y∣=1 (and $\delta_{j,k,x,y}=0$ otherwise). In order to generate emergent meta‐communities composed of species with a range of more than 1–2 patches, while preventing homogenization of the spatial network, we combine two dispersal mechanisms previously used (Abernethy, 2021), so that a small portion of each local population is constantly attempting to enter the neighboring patches, but much greater emigration will occur from patches where that population has declined since the previous ecological time step, with the fraction of the remaining population that emigrates proportional to the relative loss suffered. This is given by:

(3) $$\begin{array}{c} \mu_{i,j,k,x,y}=D_{\left(j,k\right)}^{-1}\times \frac{s_{i}}{s_{0}}\times \left\{\begin{array}{cc} \max\left\{0.001,\;0.03\left(N_{i,j,k}^{t-1}-N_{i,j,k}^{t}\right)/N_{i,j,k}^{t-1}\right\} & \text{if}\;N_{i,j,k}^{t-1}>N_{i,j,k}^{t},\\ 0.001 & \text{otherwise}. \end{array}\right. \end{array}$$

where degree $D_{\left(j,k\right)}$ is the total number of adjacent patches that can be accessed from patch $\left(j,k\right)$. Initially, this takes an integer value between 2 and 4, as we do not allow the edges of the spatial network to wrap around, and it may decrease as patches are removed during perturbation experiments. The minimum and maximum rates of movement are proportional to the bodysize of the species. Recall that the bodysize of the initial species is $s_{0}=\exp\left(1\right)$, so for a species with the mean bodysize of 3.69 (Table 1), a maximum of 4% can emigrate while for a species of the largest recorded bodysize 12.64, up to 13.9% can migrate at any one ecological time step. All species have a further restriction that a population size of at least one is required for any movement to occur.

**TABLE 1:** Initial distribution of species‐level properties of the meta‐communities

Species property	Minimum	Mean ± SD	Maximum
Range	1	1.57 ± 1.12	6
Biomass	2.93	4343.26 ± 5681.36	83353.64
Population	1.08	1327.53 ± 1679.84	22106.03
SCTL (weighted by population)	0.99	1.49 ± 0.65	4
Bodysize	0.81	3.69 ± 1.37	12.64

### 2.3 Foraging time step

Each foraging time step consists of two parts. First, the local population of each species i in patch $\left(x,y\right)$ updates their foraging efforts, focusing on the local population of species j according to:

(4) $$f_{i,j,x,y}=\frac{g_{i,j,x,y}}{\sum_{k\in K_{i}}g_{i,k,x,y}}$$

Second, they update their ratio‐dependent functional responses:

(5) $$g_{i,j,x,y}=\frac{S_{i,j}f_{i,j,x,y}N_{j,x,y}}{0.005N_{j,x,y}+\sum_{k\in P_{j}}\alpha_{i,k}S_{k,j}f_{k,j,x,y}N_{k,x,y}}$$

where $K_{i}$ and $P_{i}$ are the sets of possible prey and predator species, respectively, of species i. Competitive effects of local populations of species i and k when feeding on the same prey are controlled by their degree of similarity:

(6) $$\alpha_{i,k}=a_{1}\left(a_{2}+\left(1-a_{2}\right)q_{i,k}\exp\left(\frac{-{\left(\ln\left(s_{i}\right)-\ln\left(s_{k}\right)\right)}^{2}}{a_{3}}\right)\right)$$

where $q_{i,k}\in \left\{0,\ldots ,10\right\}$ is the number of traits shared by both species. $\alpha_{i,k}$ is designed to ensure that intraspecific competition is the strongest, and interspecific competition between species sharing a food source decreases with increasing differences between their bodysizes (increasing the index of the exponential decay function) and as they share fewer discrete traits (linearly decreasing $q_{i,k}$). In this case, $a_{1}=0.866$, $a_{2}=0.6a_{1}^{-1}$, and $a_{3}=0.6\sqrt{2\pi }$ parameterize the competition kernel in the range $\left[0.6,a_{1}\right]$.

The feeding score of species i on species j is given by:

(7) $$S_{i,j}=\max\left\{0,\sum_{m=1}^{10}\sum_{n=1}^{10}\beta_{i_{m},j_{n}}b_{1}\exp\left(\frac{-{\left(\ln\left(s_{i}\right)-\ln\left(s_{j}\right)-3\right)}^{2}}{b_{2}}\right)\right\}$$

where $i_{m}$ and $j_{n}$ are the $m^{\mathrm{th}}$ and $n^{\mathrm{th}}$ (of 10) traits of species i and j, respectively, and $b_{1}={\left(1.5\sqrt{2\pi }\right)}^{-1}$ and $b_{2}=2{\left(1.5\right)}^{2}$ scale the feeding scores kernel. The feeding scores are similarly dependent both on the traits of the predator and prey species, and on their relative bodysizes due to the exponential decay function with an optimal feeding window on species with bodysize three orders of magnitude lower than the consumer.

### 2.4 Application and limitations

Trait‐based eco‐evolutionary models (Allhoff, Ritterskamp, et al., 2015; Drossel et al., 2001; Loeuille & Loreau, 2005) typically emphasize abstraction to model general evolutionary and population dynamics mechanisms and generate plausible food web structures without describing any specific real‐world ecosystems, species, or quantifying the advantages of phenotypic traits. However, our spatial mechanics do involve some assumptions. Particularly, populations within each patch can fully “see” each other, feed and reproduce before any migration to other patches take place—indicating that patches represent a physically large environment relative to the movement range of species. Furthermore, populations may only migrate to neighboring patches and so it is only through sufficient mass effects that a population may be able to reach a suitable patch that is two steps away in the spatial network if the connecting patch cannot sustain it. Therefore, this model currently could not represent far‐ranging top predator species or migratory birds. Like other meta‐population and individual‐based studies (Furness et al., 2021), this model would therefore be most suited to describing communities of sessile species in aquatic food webs that slowly disperse across neighboring environments, or communities of low‐range vegetation and insect populations. Allometric size–structure, as implemented in this model, is also observed in both aquatic and soil food webs (Berg et al., 2015). Alternatively, meta‐population models with low rates of movement between neighboring patches are suitable for studying the interplay between the loosely connected ecosystems of island archipelagos where resident populations cannot easily move between environments on the same time scale as foraging behavior.

As simpler versions of the model have been previously studied in detail, we do not replicate investigations of the role of parameters such as c and λ, and refer the reader to those studies (Abernethy et al., 2019a; Drossel et al., 2001; Lugo & McKane, 2008). The choice of parameters used here was found to recapture non‐trivial food web structure and are not intended to predict a specific ecosystem.

Finally, in this study, we consider the extremely limiting case of a highly heterogeneous spatial network, as every patch contains a unique resource (although the resources, like species, possess 10 randomly drawn traits and some pairs of resources will therefore be more similar than others). However, future studies should investigate a mosaic of a fixed number of habitats (resources) of variable quality, in order to represent landscape patterns consisting of a meaningful spatial arrangement of several discrete habitat archetypes. This would facilitate investigations of the impact of the autocorrelation of habitat types and the use of wildlife corridors for restoring or converting habitats.

## 3 META‐COMMUNITY PROPERTIES

We begin with an overview of the meta‐communities, each generated by sequences of 10,000 evolutionary timesteps in the model described in §2. This results in 540 coupled communities of between 6 and 36 species, arranged in 15 meta‐communities of two‐dimensional 6×6 spatial lattices. For these statistics, the data from all the ensembles are gathered together so that averages are taken over the entire set of communities and species.

### 3.1 Community properties

Global diversity (including 36 resources) in each meta‐community varied between 465 and 546. The distribution of community properties across the 540 patches is shown in Table 2.

**TABLE 2:** Distribution of patch‐level properties of the meta‐communities

Patch property	Minimum	Mean ± SD	Maximum
Local diversity	7	21.47 ± 4.53	37
True local diversity	2	17.23 ± 4.62	32
Link density	1	2.24 ± 0.30	3.15
Connectance	0.15	0.23 ± 0.04	0.53
Average SCTL	1	1.64 ± 0.23	2.31
Max SCTL	1	3.04 ± 0.62	5
Omnivory fraction	0.1	0.34 ± 0.09	0.65
Average population	417.55	886.11 ± 272.43	3068.27
Average bodysize	2.52	3.70 ± 0.61	6.56
Average range	1.65	2.43 ± 0.41	4.6

**FIGURE 1:**
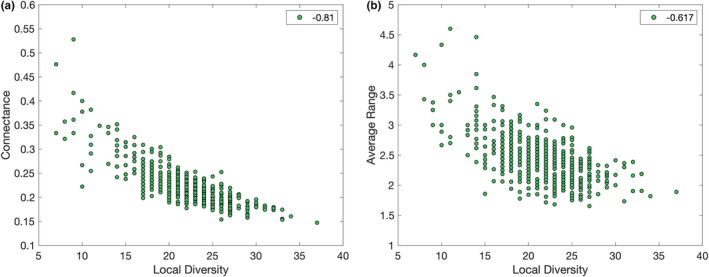
Selected initial patch‐level properties

Describing the structure of the community, local diversity refers to the number of species (including resources) in a patch, which reduces to the “true” local diversity when isolated from the rest of the meta‐community and any satellite populations perish. Link density is the ratio of feeding relationships to species, and connectance is the fraction of all possible feeding links in the food web that are realized. For average properties of the resident populations in each community, the trophic level (SCTL) of a local population is calculated as the shortest length of food chain from the species to the resource. Species that feed on at least two others in the same community with different shortest‐chain trophic levels are counted as omnivores. The range of a species is the number of patches out of the 36 in the meta‐community that it is resident in with non‐trivial population. Due to the heterogeneous habitats and nearest‐neighbor movement described in §2.4, species range remains relatively low and the coupling between patches not strong. Consequently, although central patches had the lowest diversity, the variation was small and no strong dependencies were observed between the properties of a patch's ecosystem and its position within the regular lattice meta‐network, in contrast to the trends observed in other more complex spatial networks (Cuddington & Yodzis, 2002). The relationships between structural properties of the local food webs within patches are in agreement with those previously demonstrated (Abernethy et al., 2019a) for non‐spatial single‐community versions of this model. In particular, connectance decreases with local biodiversity (Figure 1a).

Calculating Pearson correlation coefficients, a higher diversity is associated with increased link density (0.71) and average trophic levels (0.33), but reduced connectance (−0.81) and average population (−0.71), range (−0.62 and see Figure 1b), and bodysize (−0.25) of the resident species. Larger species tend to occupy higher trophic levels, but bodysize has no direct relationship with range (see §3.2). Thus, high‐diversity communities can accommodate some higher‐level species, raising the average and maximum trophic levels compared to lower‐diversity ecosystems which may only have one trophic level of species that directly consume the resource. However, the majority of additional species will be specialized basal or primary consumers with low populations that experience significant competition. Negative correlations exist between diversity and range because as the local community co‐evolves and increases in diversity, specialists better adapted to exploit that particular patch (thus, with low range) are more likely to arise and to exclude less well‐adapted generalists from neighboring patches.

### 3.2 Species properties

Next, consider the distribution of the properties of the 7022 species present in the meta‐communities.

**FIGURE 2:**
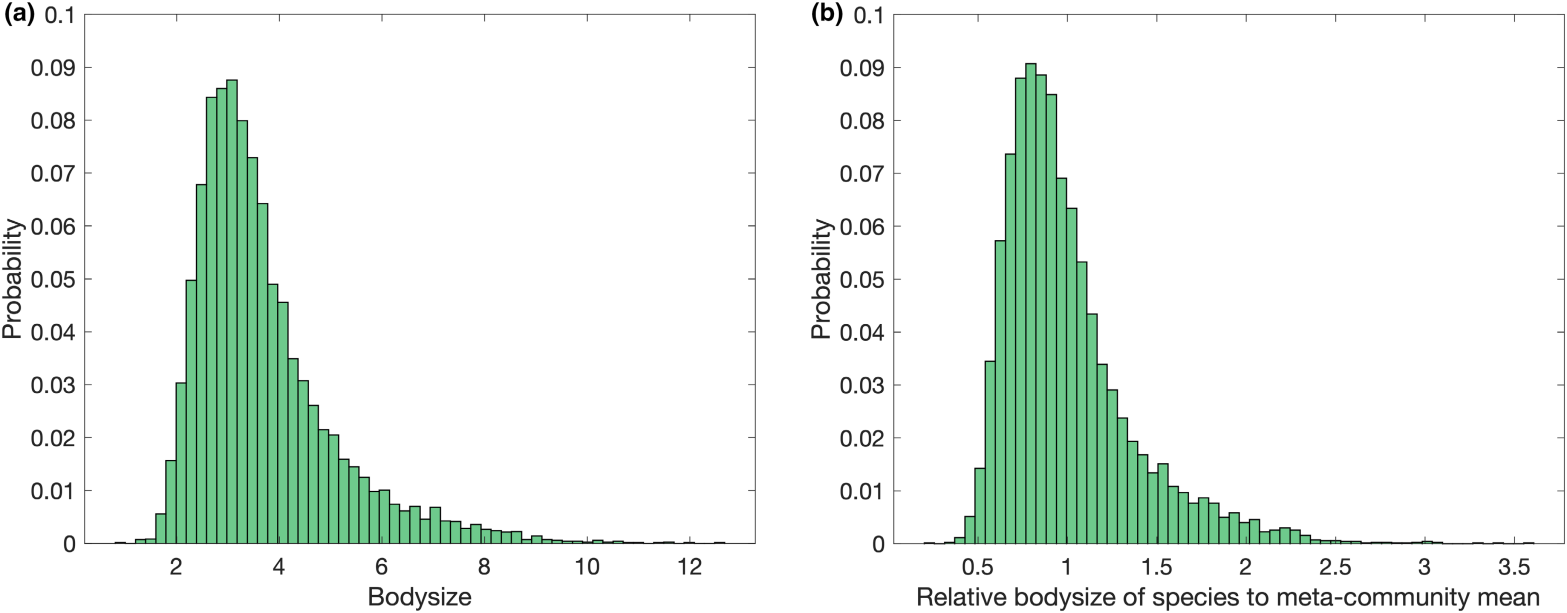
Distribution of species' bodysize

The distribution of the bodysize of the species is illustrated in Figure 2. As anticipated by the model design, a mild positive correlation (Pearson correlation coefficient of 0.2) is observed between trophic level and bodysize, as larger bodysize predisposes species to higher trophic levels. Close to bodysize of 2.0, the majority of species transition from level 1 to level 2. Moderate negative correlations are seen between population or biomass and measures of trophic level due to ecological efficiency. Consistently no relationship was observed between bodysize and range due to the trade‐off between the increased mobility of larger species' and their requirement for additional resources. At higher trophic levels, large‐bodied species may not experience sufficient predation pressure to trigger emigration.

The majority of local sub‐populations of species occupy low trophic levels as this allows access to the most resource biomass due to ecological efficiency, and this is especially the case in low‐diversity communities. A species' trophic role is not fixed across patches, so a far‐ranging species has additional opportunities to exploit low trophic levels. In these patches, the species will be able to maintain a larger local population, resulting in a reduced shortest‐chain trophic level averaged over all its sub‐populations. Consequently, a negative correlation between SCTL and range (−0.31) was observed. Unsurprisingly, the range was also strongly positively correlated with absolute biomass (0.73) and population (0.72) as occupying additional patches multiplies the opportunities for population growth and the as‐described greater range results in increased opportunities to directly exploit a resource and thus supports large, low‐level populations.

### 3.3 Species–area relationship

**FIGURE 3:**
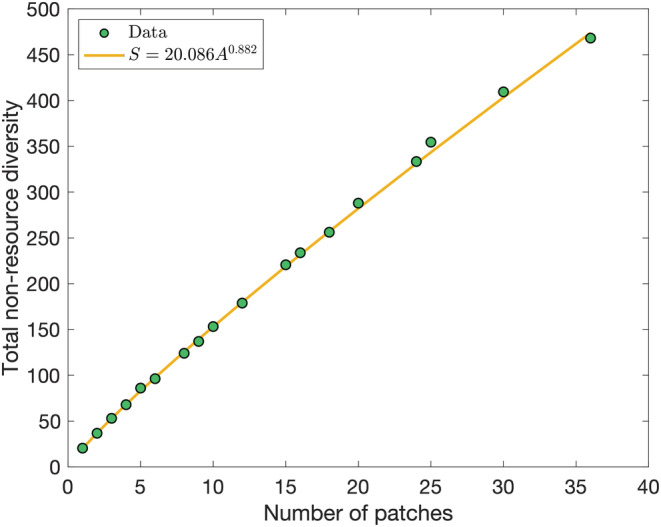
Species–area relationship

To quantify the range and distribution of species, we calculate the species–area curve. For each rectangular area that it is possible to draw in a 6×6 grid (i.e., for each of the 18 sizes that arise from combinations of i×j for $i,j\in \left\{1,2,3,4,5,6\right\}$ patches), we calculate the mean γ‐diversity (total species present) across every possible rectangular subset of that many patches across each meta‐community. This is shown in Figure 3 along with the best‐fit power‐law relationship between the area of A patches and diversity S.

Although species display greater range than previous simulations with this model without resulting in homogenization, the species–area exponent of 0.882 remains much larger than realistic estimates (around 0.25–0.27 (Drakare et al., 2006; Pelletier, 1999)) for the large‐scale distribution of species. This is due to the unique resources in each patch as discussed in §2.4, which combined with the nearest‐neighbor migration mechanism and the competition function causes species sorting as the model selects for specialists in each community. However, it is not dissimilar from power laws observed (0.59–0.65 dependent on migration rates) with fewer than 100 patches in a similar three‐trait meta‐community model (Rogge et al., 2018). While future model communities should aim for a smaller exponent in order to be applicable to terrestrial ecosystems, the following results will be relevant to “large world” scenarios such as connecting distinct and geographically separated ecosystems by shipping and human transportation.

## 4 SPECIES DELETION

For each of the ensembles, we conduct an in silico experiment on the effect on the meta‐community of systematically deleting each species and iterating the ecological loop allowing the remaining populations to adapt their feeding choices, reproduce, and potentially disperse to neighboring patches. The impact of this primary extinction is recorded and the system is subsequently reset to its original pre‐perturbation state before the next species is deleted. Such perturbation studies have been conducted on food webs constructed from empirical lakes (Thara Srinivasan et al., 2007), using (Berg et al., 2015) community models with Lotka–Volterra dynamics and using adaptive dynamics in non‐spatial configurations of the Webworld model (Quince et al., 2005).

From Pearson correlation coefficients, the complexity and diversity of spatial meta‐communities have eliminated the weak relationships previously observed between secondary extinctions and trophic level or bodysize (Abernethy, 2020) or that the loss of species on extreme trophic levels was more disruptive. (Quince et al., 2005). However, a weak relationship is seen between relative secondary extinctions and the species' range (0.154), and with it a correlation with population (0.139) and biomass (0.143) that was *not* present in the non‐spatial version of this model when allometric effects were included.

Overall, the percentages of secondary extinctions are extremely small due to the weak coupling between communities resulting in a very strong stability against the loss of any one species. Absolute secondary extinctions range from 0 to 10 with a mean of 0.67, while relative secondary extinctions range from 0 to 0.0206 with a mean of 0.0014. Previous non‐spatial species deletion studies observed an average of 2.1% of remaining species lost (Quince et al., 2005) and a maximum of 17 secondary extinctions in single ecosystems that comprised 64 species on average. The meta‐community ensembles of over 400 species tested here never lost more than 10 due to a single primary extinction, despite the robustness of individual communities being known to decrease with increasing diversity (Abernethy et al., 2019a). However, it would be too simple to state that spatial effects are yielding greater stability, as if the 36 patches in each meta‐community were to be fully uncoupled they could each potentially host large food webs—but this would require an initial species and 10,000 speciation events per each community rather than one of each overall. In a meta‐community of this size (36 patches and 400–500 species) and high SAR exponent, the specific role of any one species in the few patches that they occupy is outweighed by how few local ecosystems the loss of this species will directly affect, with the magnitude of that effect being encapsulated in the biomass or population size rather than trophic level or bodysize. Multiple linear regression was used to determine if secondary extinctions can be predicted by any combination of species properties, however, the best adjusted‐*R* ^2^ was only 0.03 and species range gives the best sole‐predictor result with just 0.02.

Finally, for comparison with previous investigations (Drossel et al., 2001), consider the functions describing the frequency distribution of the size of extinction events due to the deletion of the species. This is of interest, as evidence for power‐law (rather than exponential) distributions could indicate the potential for self‐organized criticality, and thus the possibility of arbitrarily large extinction events to occur in the ecosystem if the simulation is allowed to execute for a sufficient amount of runtime as speciation events trigger the loss of a pre‐established species which themselves precipitate an extinction cascade. For both absolute and relative secondary extinction event sizes, both a power and an exponential decay law can be fitted with $R^{2}$ between 0.96 and 0.97. While this is not conclusive, the small maximal extinction events with non‐zero frequency and the large fitted indices suggest that massive cascades are not likely given how the distinct resource types and low species range results in an effective large‐world structure of the spatial network with strong species sorting effects. This creates a buffering effect that prevents localized shocks (the unexpected loss of a single species present in 2–3 patches only) from propagating through the entire global network.

## 5 SPECIES INVASION

In addition to the elimination of species resulting directly from anthropogenic activity, extinctions resulting indirectly from the invasion of exotic species displaced or introduced by humans are a significant concern (Bellard et al., 2016). Following the previous experiment in §4, after each species is deleted in isolation, it is re‐introduced to every patch in its associated meta‐community (rather than just those patches that it inhabited prior to its forced extinction) with the minimum sub‐population size of 1.0. The ecological loop is again iterated and we subsequently record alterations to the meta‐community properties, in particular the number of secondary extinctions that further result from the re‐introduction of the species to all patches, and the number of communities (of 36) that it successfully invades.

**FIGURE 4:**
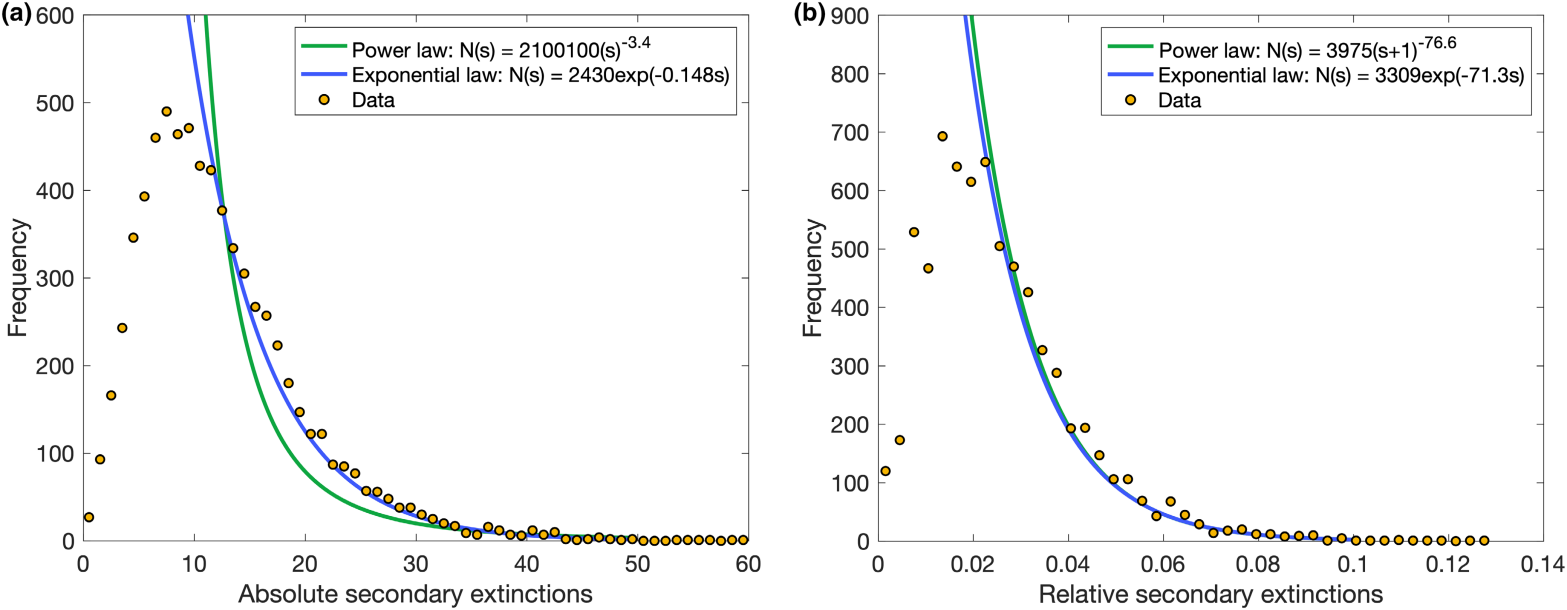
Distribution of size of 7022 extinction events caused by re‐introduction of an eliminated species to all patches in its meta‐community

Absolute secondary extinctions range from 0 to 60 with a mean of 11.80, while relative extinctions (as a fraction of the species in the meta‐community, excluding the invader) range from 0 to 0.1288 with a mean of 0.0252. As in §4, we investigate the frequency distribution of the size of the secondary extinction events resulting from invasion, with each decay model (illustrated in Figure 4) fitted from the peak frequency bin to the final non‐zero frequency bin of the distribution. For absolute extinction events sizes, the power law is fitted to the linear log–log graph with $R^{2}=0.88$, while the exponential relationship gives a better fit $R^{2}=0.96$ to the semi‐log plot. For relative extinctions, both decay laws fit $R^{2}=0.97$. Previous studies of the size distribution of extinction events in each species introduction in a single patch obtained evidence of exponential decay (Drossel et al., 2001) or a very slight preference for an exponential over a power law (Abernethy et al., 2019a). Here, we recover a stronger argument for exponential decay, indicating that arbitrarily large absolute extinction events in a trophic meta‐community are improbable. This is not surprising given the resource‐imposed limitations on the maximum number of species and given that not every community will be invadable to every species. Considering instead the relative impact on the ecosystem, there is no preference between the proposed decay laws, and as more than 12% of a mature meta‐community can be destroyed merely by increasing the mobility of a species that already exists within the spatial network, it remains possible that an even larger fraction of biodiversity could be wiped out by the displacement of a single species to new patches within its meta‐community. This differs from the result of single species deletion in §4, as the perturbation simultaneously affects the entire global meta‐community when the species is introduced to all patches—effectively side‐stepping the spatial containment of perturbations previously observed that large functional separations in space provide and which facilitate co‐existence of competitors (Barter & Gross, 2017) for the same trophic niches. While this is unlikely to represent an event that could occur naturally where a species that has evolved in a localized region is suddenly able to disperse itself much farther than previously possible, human activity can create corridors between otherwise far separated environments (whether intentionally through misjudged conservation efforts or simply by the establishment of transportation networks), and this experiment demonstrates that minimal populations could be sufficient to damage regional biodiversity in such circumstances.

To determine whether the impact of the invasion by a particular species can be predicted according to its trophic role, correlation coefficients between properties of each of the 7022 non‐resource species, its ability to re‐invade the meta‐network, and the subsequent negative impact on biodiversity are shown in Table 3. Relative extinction event sizes are moderately positively correlated with the invader bodysize, as this property predicts the number of patches invaded (the strongest correlation is unsurprisingly between this outcome and the resulting extinctions). Increased invasion success for large‐bodied species with greater dispersal rates has been observed in other meta‐community invasion models (Häussler et al., 2021), and this does not seem dependent on the prior range of the species. In §3.2, we did not find an initial correlation between bodysize and range (Table 3), although as large species maintain smaller populations they may have especially benefited from re‐introductions with uniform populations. This demonstrates the ability of high‐bodysize species to establish themselves and impact ecosystems only if an artificial intervention introduces them to areas beyond their natural bounds in the global meta‐community. Multiple linear regression confirms that the prediction of extinctions using bodysize can be improved by considering the trophic level of the invader, while the number of patches invaded can be predicted with moderate accuracy by bodysize alone with slight improvement by including the original range of the species.

**TABLE 3:** Pearson correlation coefficient of initial species‐level properties with the outcome of their re‐introduction to the entire meta‐network

Species property	Number of successful invasions	Relative secondary extinctions
Range	0.069 (−0.020 to 0.173)	−0.016 (−0.107 to 0.115)
Biomass	0.015 (−0.076 to 0.159)	−0.034 (−0.178 to 0.167)
Population	−0.127 (−0.205 to −0.034)	−0.173 (−0.276 to −0.062)
SCTL (weighted by population)	0.201 (0.075 to 0.331)	0.186 (0.025 to 0.292)
Bodysize	0.566 (0.499 to 0.675)	0.622 (0.537 to 0.762)

**FIGURE 5:**
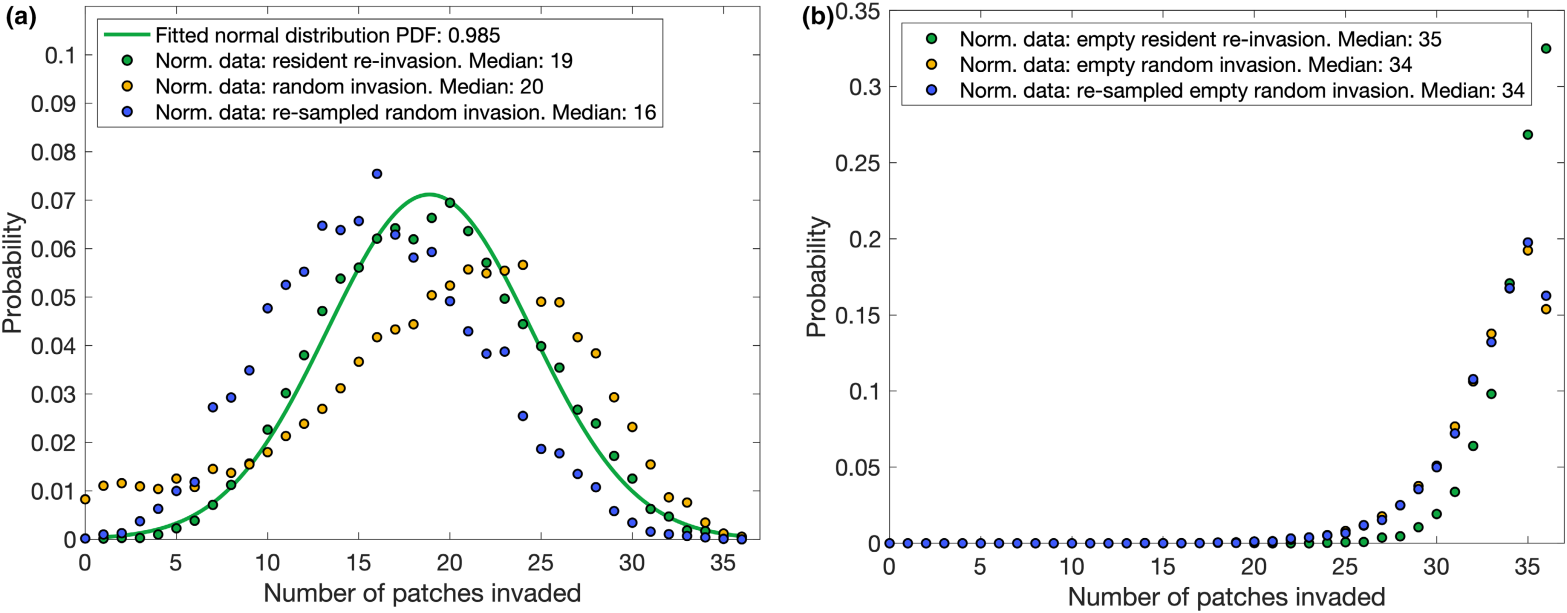
Probability distribution of the number of patches invaded by species introduced to the entire (a) occupied and (b) empty meta‐network

The distribution of the number of patches successfully inhabited following invasion covers the full range from 1 (the species is always able to re‐enter at least one habitat that it was previously present in) to 36, with a mean of 18.88 and a standard deviation of 5.60. Fitting the normalized frequency distribution (with re‐scaling parameter 0.000144) to a normal distribution with the same parameters yields an $R^{2}$ value of 0.99 (Figure 5a, green). This is surprising, as species had a maximum prior range of 1/6 of the network (Table 1), while this experiment suggests a random species and patch from its meta‐network has an expected 50% chance to successfully invade, and furthermore, a non‐zero number of species successfully occupied *every* patch. There must therefore exist species that can persist in the communities of all patches of their meta‐community, yet fail to generate sufficient local populations to conduct the necessary invasions without artificial assistance.

If the meta‐network is cleared of all species in a global extinction event, and each species is individually re‐introduced in isolation from all others (Figure 5b, green), they are able to invade a minimum of 20 patches and more than half can invade 35–36 patches. However, many of these are due to mass effects and source–sink dynamics with large sustainable local populations maintaining satellite populations in neighboring patches that are not themselves sustainable without this support. A random species has a probability of exactly 0.5 (due to the β scores matrix) of being able to consume any given resource and so the expected number of invasions in a totally disconnected spatial network is 18 patches. For comparison, for each meta‐community, we generate a set of 500 species (7500 in total) with random traits and bodysize drawn from a uniform distribution across the observed range (0.81–12.64), and similarly introduce them to all patches in the pre‐existing meta‐communities. Likewise, we simulate the arrival of 2000 species (30,000 in total) to each empty meta‐network (Figure 5, yellow). To distinguish the influence of bodysize distribution and co‐evolved traits, we then drew 20,000 re‐samples of sizes 800 and 5000, respectively, from these random species sets according to the actual absolute bodysize distribution (Figure 2a) of the combined meta‐communities. The mean probability distribution of the number of patches invaded over these re‐samples is also illustrated (Figure 5, blue).

**FIGURE 6:**
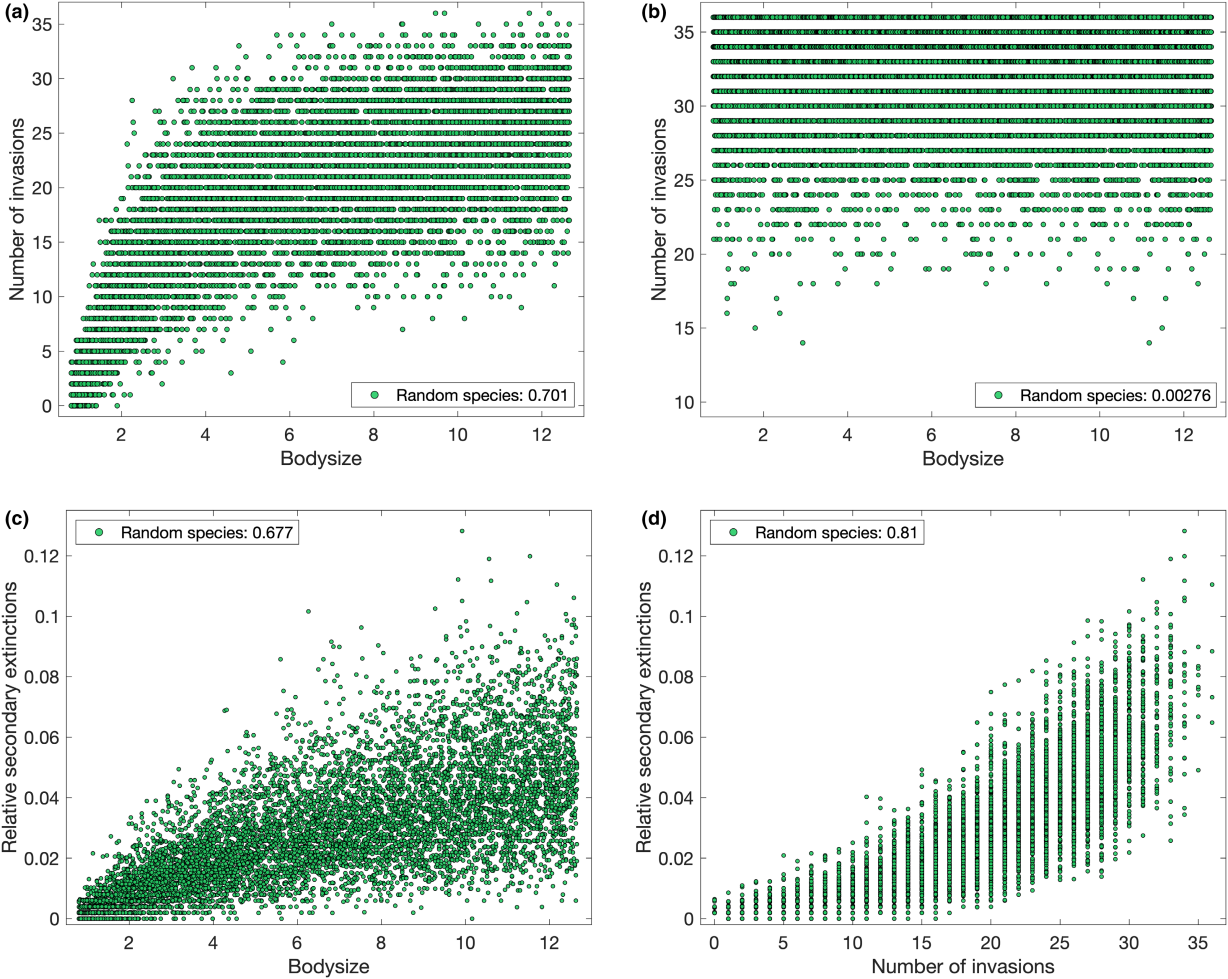
Outcomes of invasion of (a, c, d) occupied and (b) empty meta‐networks by randomly generated species with uniformly distributed bodysize

**FIGURE 7:**
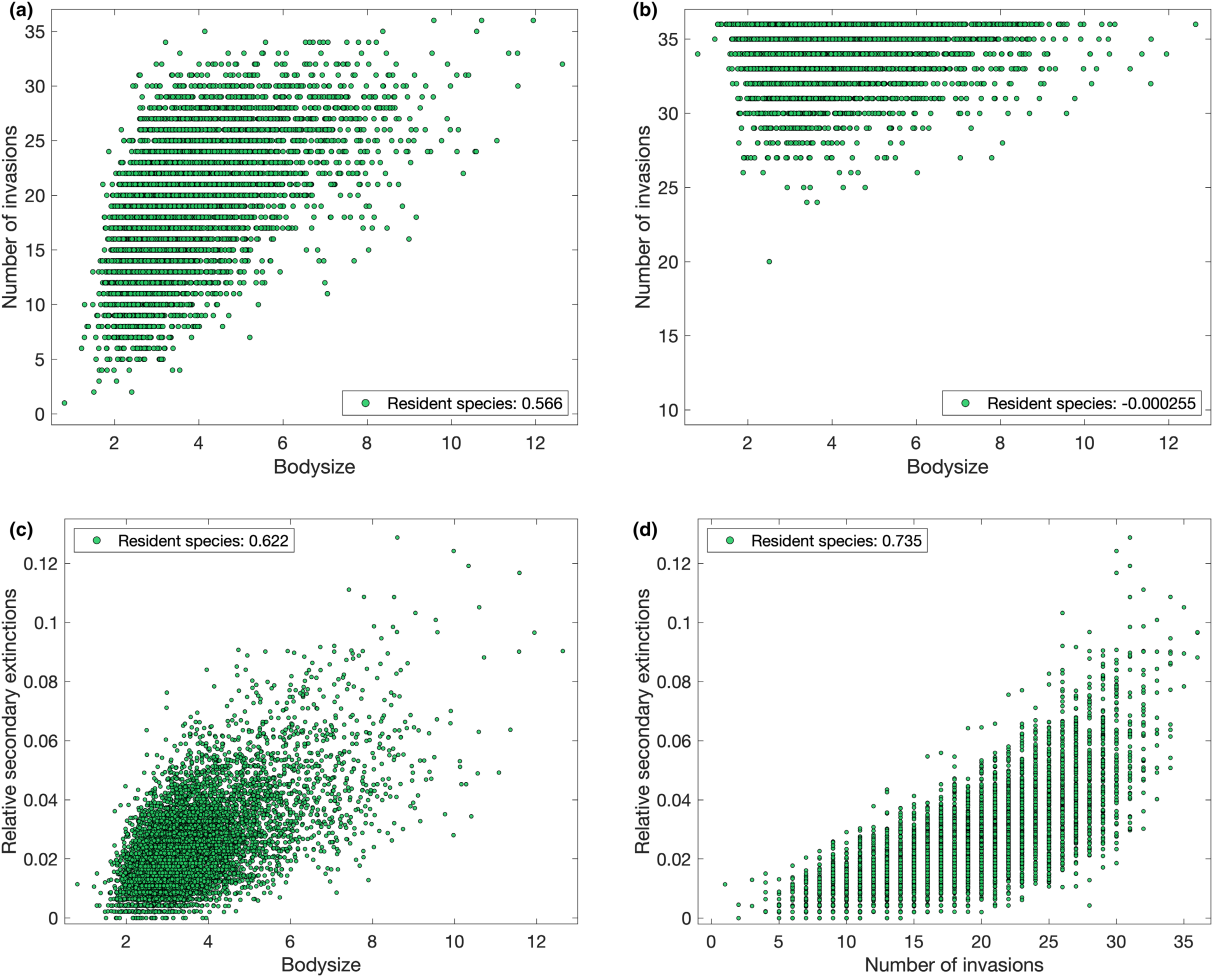
Outcomes of invasion by a single resident species

Randomly generated species with uniform bodysize distribution have an increased chance of successful invasions of the occupied meta‐communities (Figure 5a, yellow), accompanied by an increased risk of few or zero patches invaded. The latter effect is due to species with very low bodysizes (0.8–2.0) (Figure 6a), only a small number of which would be present in the original distribution (compared with Figure 7a and see also Figure 2a). To determine if the overall increased invasion probability is due to bodysize, consider the re‐sampled set (Figure 5a, blue). In this case, the mean correlation coefficient with bodysize is still positive (0.502, with 95% confidence interval 0.501–0.502), although reduced in comparison to resident species (0.566). When bodysize distribution is controlled, we recover a very low probability of invading zero to five patches, and return to an overall invasion advantage for the co‐evolved species. Such species have already had their traits selected for (and some may be effective consumers of one or more resources), and their previous existence in the meta‐community over evolutionary time scales may have influenced its subsequent development and what other species were able to establish through priority effects (Weidlich et al., 2021), both of these mechanisms will confer benefits to invasion success. Thus, the increased invasion chance for random species with uniform bodysize distribution was due to the presence of so many large bodysize species with high invasion probability over‐compensating for the disadvantage of lacking a co‐evolved selection of traits. In contrast, when introduced to an empty network, bodysize ceases to be influential with the resident species, uniformly distributed bodysize random species, and the re‐sampled bodysize random species all yielding correlation coefficients under 0.02 between the bodysize and the number of patches occupied. Instead, note that co‐evolved species are more likely to successfully invade every patch (Figure 5b, green). Thus, co‐evolution impacts invasion probability in empty networks to an extent, while bodysize does not as, although large bodysize will reduce the effectiveness of feeding on resources, it will never prevent it in this model.

## 6 INDIVIDUAL PATCH DELETION

In addition to altering the composition of species, spatially explicit meta‐population models can simulate the effects of environmental challenges through patch fragmentation and removal. Recent work in single‐species meta‐population theory has demonstrated that the relative importance of a given patch for migratory species can depend on the severity of this perturbation (Sample et al., 2020). Similarly, empirical studies have shown that the severity of perturbation can determine the speed and composition of recovering communities (Davies et al., 1999) and that the assumptions encoded about species re‐settlement behavior can crucially impact the accuracy of predictions (Burns & Grear, 2008). Therefore, in this section, we undertake four kinds of perturbation of individual patch communities. First, it may be either a temporary “pulse‐perturbation” where populations from other patches are permitted to re‐colonize the patch following the disruption, or a permanent “press‐perturbation” where they are not. Second, the immediate consequence may be either elimination of the residents' populations, or displacement where they are evenly distributed among all neighboring patches. As with regular migration in the model, neighboring patches are considered to be the 2–4 (non‐diagonally) adjacent patches. This set of experiments was previously tested for two ensembles with reduced species range resulting from different dispersal rules (Abernethy, 2021), and in a set of models that were compared with the observations of empirical experiments on white‐footed mice (Burns & Grear, 2008). Permanent patch destruction models urban re‐development of formerly natural sites by the building of houses, roads, and utilities, while temporary destruction represents the transient effects of floods, wildfires, or managed industrial logging and forestry practices.

As a consequence of perturbing a single patch in the meta‐communities, the relative loss of global diversity ranges from −0.054 to 0 (no patch hosted zero species, so zero extinctions can occur when disrupting a patch whose resident species all also existed elsewhere in the meta‐community) for temporary elimination, −0.054 to −0.002 for temporary displacement, −0.051 to −0.002 for permanent elimination, and −0.065 to −0.004 for permanent displacement. Thus, displacement can cause the greatest harm (over 6%) to a meta‐community. The number of surviving resident species tended to be larger for the temporary than permanent disruption, indicating that some displaced populations re‐colonize the disrupted habitat if they are not prevented from doing so. If this happens, both more species unique to the original patch (hereafter “unique residents”) and more species unique to the neighboring patches (i.e., not present in the perturbed patch—hereafter “unique neighbors”) survive. Thus, if prevented from re‐entering their home patch, most displaced residents will perish along with some of the original neighbors that resided in invaded patches prior to the disturbance.

When patch contents are eliminated, global biodiversity loss strongly correlates with the number of resident species (Pearson correlation coefficient 0.75 and 0.74 for temporary and permanent perturbation, respectively) and especially with the number of unique residents (0.84 and 0.88). Diversity loss also correlates with high link density, low average populations, low average biomass, and low connectance as these community properties are all associated with high local biodiversity (§3.1). There is a negative correlation with the average range of residents, as if eliminated populations belong to species with populations in other patches, then it will not go extinct from the meta‐community. A multiple linear regression model can be fitted for biodiversity loss from temporary elimination experiments with an adjusted‐*R* ^2^ of 0.72 when accounting for the number of resident species, their range, and the number of patches connected to the perturbed one, as highly connected patches are more likely to host satellite populations that will persist in their source patches. However, 0.70 can be obtained only considering the number of unique residents that will certainly not survive the perturbation. Similarly, when the elimination is permanent 0.77 is obtained just from the number of unique residents. When the perturbation displaces residents into neighboring patches, high‐range species remain less likely to go extinct if their displaced local populations fail to invade neighboring patches, and they may already be present in the neighboring patches reducing the disruptive effects of the invasion. In isolation, there is no correlation between relative global biodiversity loss and the average bodysize of (unique) residents or the ratio between the average bodysize of the unique residents to that of unique neighbors. However, these properties become non‐trivial when combined with others in a multiple linear regression model. These yielded adjusted‐*R* ^2^ values of 0.29 and 0.26 for temporary and permanent displacement, respectively, and 0.23 can be achieved solely using the number of unique residents. This is improved by accounting for the ratio of unique residents to unique neighbors, and either the mean bodysize of unique residents or the ratio of mean resident to neighbor bodysizes (larger resident species are more likely to invade successfully, §5). Thus, when the residents of a patch are displaced, global diversity loss is less dependent on the survivability of the evicted residents than how much impact they may have on neighboring communities. This is quantified by the number of invaders (unique residents) and their bodysize to predict their potential for invasion, combined with the number of neighbors for how much loss can be suffered by the invaded patches.

**FIGURE 8:**
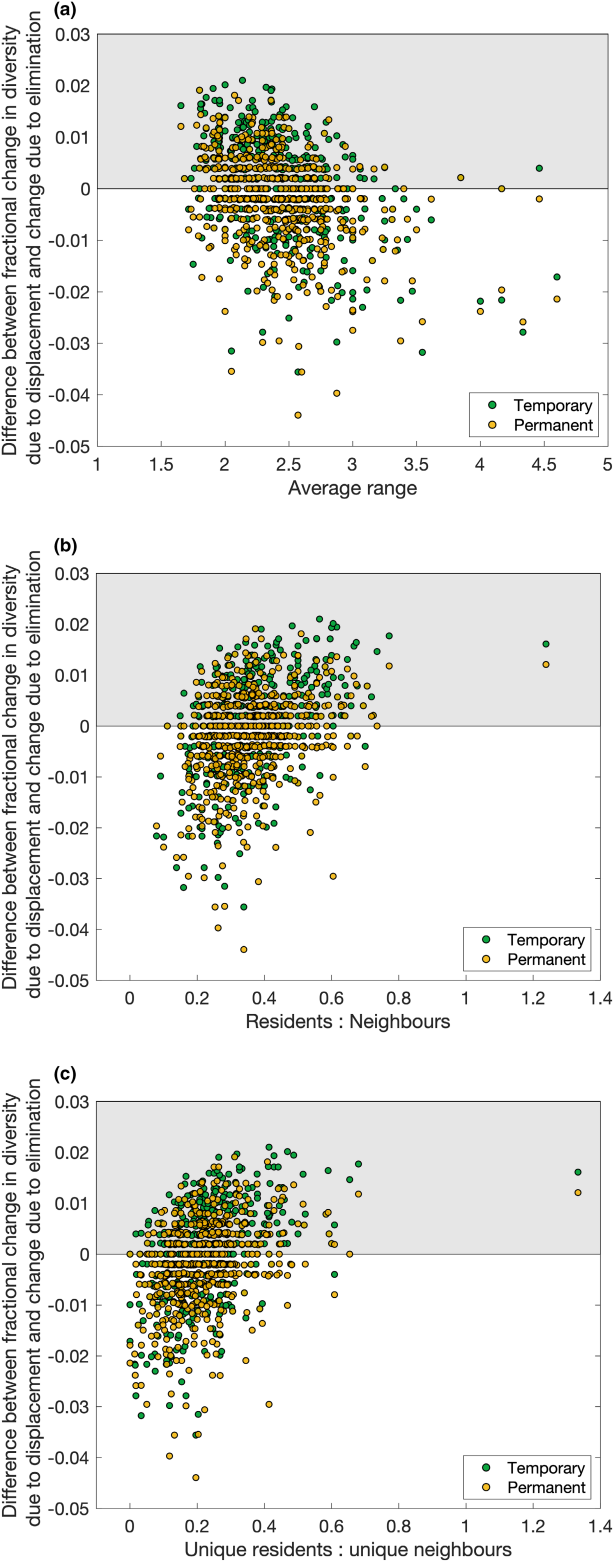
The difference between displacement and elimination of the patch

Inspired by previous results (Abernethy, 2021), we seek indicators for when the displacement of the population of a patch will be more damaging than simply eliminating them. This is a pertinent question in conservation scenarios where a unique local habitat faces destruction. For each patch, the difference between the fractional change in the global ecosystem due to displacement and elimination of the resident populations is calculated. When this net quantity is positive (shaded area of Figure 8), it is beneficial for the conservation of global biodiversity to ensure affected residents are re‐settled in neighboring patches. The strongest correlations are with the average range of the species (−0.394 and −0.347 for temporary and permanent, respectively) and the ratios of either the total or the unique numbers of residents to the number of (total or unique) neighbors (0.412 and 0.312 for the total ratios and 0.423 and 0.336 for the ratios of unique species). Thus, the question is whether the perturbed patch or its neighbors possess greater unique contributions to biodiversity and should therefore be prioritized. If the perturbed patch has relatively many unique residents rarely found elsewhere in the meta‐community, it may be better to re‐settle these populations despite possible harm to the surrounding communities. However, if the target patch has relatively few species (or they are common elsewhere) and is surrounded by flourishing neighbors, elimination is less likely to result in unexpected extinction waves. However, this assumes that populations cannot be re‐settled in a more directed manner to suitable or similar habitats, and this result may be applicable only to a heterogeneous spatial network where every patch has different environmental features.

## 7 SEQUENTIAL PATCH DISRUPTION

**FIGURE 9:**
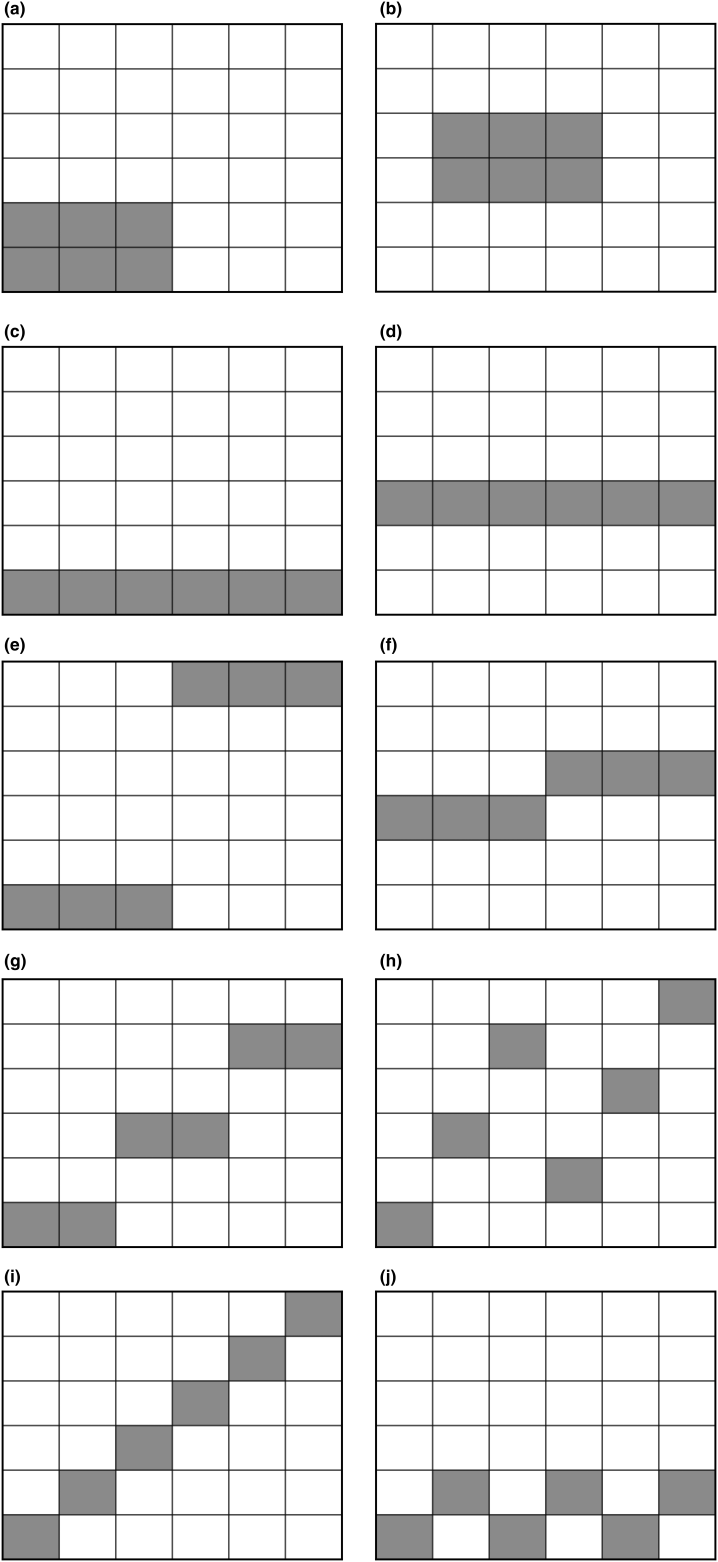
Fixed placements of nature reserves

Similar to previous studies that have considered the impact of sequences of repeated perturbation by deleting a species, we now generalize the patch deletion experiment to sequences of patch deletion. Meta‐population models have been used to test the robustness of a species or simple meta‐community to increasing habitat loss, demonstrating how the structural properties of community food webs can influence species persistence against habitat loss (Melián & Bascompte, 2002), and the possibility of non‐intuitive outcomes of dynamic mechanisms in complex meta‐communities. In some cases, limited habitat loss can increase species richness in the remaining habitats by reducing competitive exclusion effects due to specialist consumers (Pillai et al., 2011). Some models have been applied to testing reserve design and placement in space, testing the benefits of large or small but multiple reserves (Pelletier, 2000), and finding that if reserves are small enough for space to be a limitation, the resulting small population sizes may cancel out the benefits of redundancy in species diversity for preventing community collapse in the event of species loss (Ebenman et al., 2004). The final perturbation experiment here extends these investigations to explicit population dynamics of complex co‐evolved food webs in a strongly heterogeneous spatial environment. The 15 meta‐communities are subjected to repeated sequences of the perturbations introduced in §6. Six patches may be designated as reserves that are exempt from perturbation. These are selected according to 12 schemes (13 including the control scenario without reserves), 10 of which are pre‐defined and illustrated in Figure 9. The remainder are to select the six patches with the highest local diversity, or the six whose resident species have the lowest average range. The meta‐communities undergo sequences of either 1000 temporary perturbations (where each non‐reserve patch may be selected multiple times) or 30 permanent perturbations (36 if there are no reserves). In each case, the highest‐diversity patch available at that moment is targeted in one sequence, and this is compared with the average results of 10 sequences of randomly selecting the target from all available non‐reserve patches. Overall, this experiment executes 4,420,680 perturbation events in 15×4×13×11=8580 independent sequences.

**FIGURE 10:**
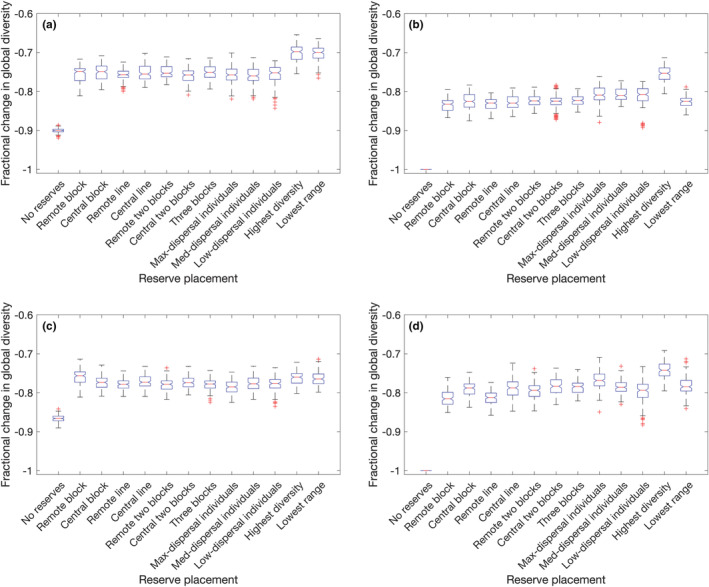
Final outcomes of random perturbation sequences: (a) temporary elimination, (b) permanent elimination, (c) temporary displacement, (d) permanent displacement.

**FIGURE 11:**
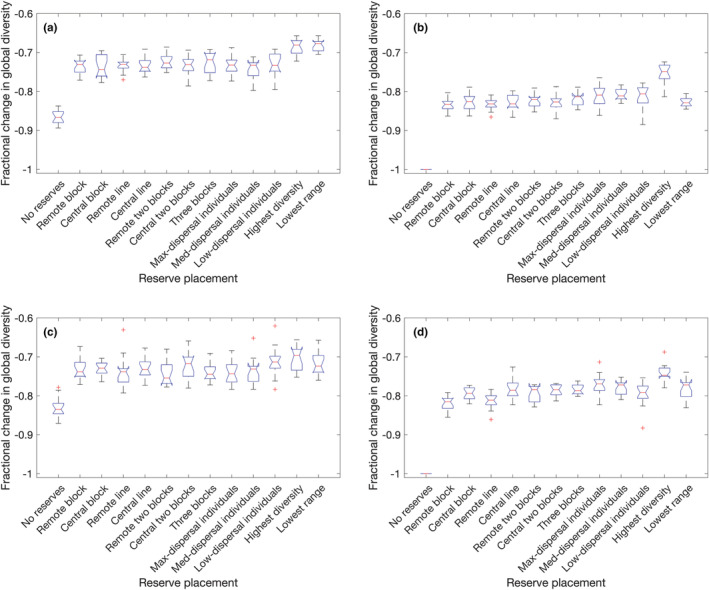
Final outcomes of targeted perturbation sequences: (a) temporary elimination, (b) permanent elimination, (c) temporary displacement, (d) permanent displacement.

The final relative biodiversity outcomes are illustrated in Figure 10 for randomly selected patch sequences and Figure 11 for targeted selection, where for each perturbation type and reserve strategy, the result is calculated over 15×10=150 or 15 sequences, respectively. If reserves are absent, repeated temporary elimination is consistently more damaging than repeated temporary displacement. Reserves targeting the highest diversity patches are uniquely the best choice when the perturbation is permanent (Figures 10 and 11b,d). ANOVA and subsequent testing reveal additional patterns: all three individual‐cell reserve placements (Figure 9h–j) are superior to other pre‐defined reserve locations in the case of random–permanent–elimination perturbations (Figure 10b), while of these, only the maximum‐dispersal individual patch reserves (Figure 9h) are also statistically superior in the case of random–permanent–displacement perturbations (Figure 10d). This is because choosing these patches is likely to maximize the number of unique residents, given that most species have a range of 2–3 patches which are likely to be neighbors. If reserves are spread far apart, this increases the chance of protecting unique species from elimination. For displacement, although the reserves will eventually be invaded by the residents of neighboring patches as permanent perturbations gradually remove alternatives, it is still preferable to protect the co‐evolved ecosystems of as many distinct species as possible so as to maximize the number of species that could ultimately survive invasions.

Against a temporary elimination, the optimal reserve placements target either the highest diversity or the lowest average range (Figures 10 and 11a). The distinction between these scenarios and permanent elimination is that there is now the possibility of immediate re‐colonization of patches that have been disrupted, so focusing protections on the species that are least able to take advantage of this recovery mechanism (due to maintaining populations in relatively few patches) is now beneficial when it would not ultimately have mattered in the permanent disruption case.

For temporary displacement sequences (Figures 10 and 11c), the advantage over pre‐defined reserve placements is less clear. Against targeted perturbation, ANOVA tests confirm that neither is significantly better. However, for random perturbation sequences, both “highest diversity” and “lowest range” reserve strategies yield a statistically significant improvement except over “remote block” reserve placement (Figure 9a) which is also advantageous with the same level of significance (*p*‐value on the order of $2.8-2.9\times 10^{-7}$). This is because when a patch's populations are displaced, it is neighboring species that are at risk of extinction as much as the evicted residents (§6), so an ideal strategy would prevent communities of rare species from being invaded, and the only pre‐defined reserve placement that guarantees that some patches will never have neighboring populations displaced directly into them is the remote block strategy which totally isolates patches (1,1) and (1,2) in the corner of the network. This block of reserves has only five input paths, while the central block has 10, and the maximum‐dispersal individual reserve placements have 20, so in addition to fully isolating some patches, this strategy also minimizes the number of invasions that its other protected patches will undergo during the experiment. This suggests that there is a benefit to situating nature reserves in remote, isolated regions so that they are protected not just from direct development but also from the disruptive effects of nearby habitat destruction. As with permanent displacement, it is still also beneficial to designate high‐diversity patches as reserves, as although these species are not sheltered from invasion, it does at least protect the greatest number of species from being themselves displaced and thus perishing or causing extinctions. Finally, protecting rare species means that those which are displaced will be more likely to already be present in the neighboring patch they invade.

**FIGURE 12:**
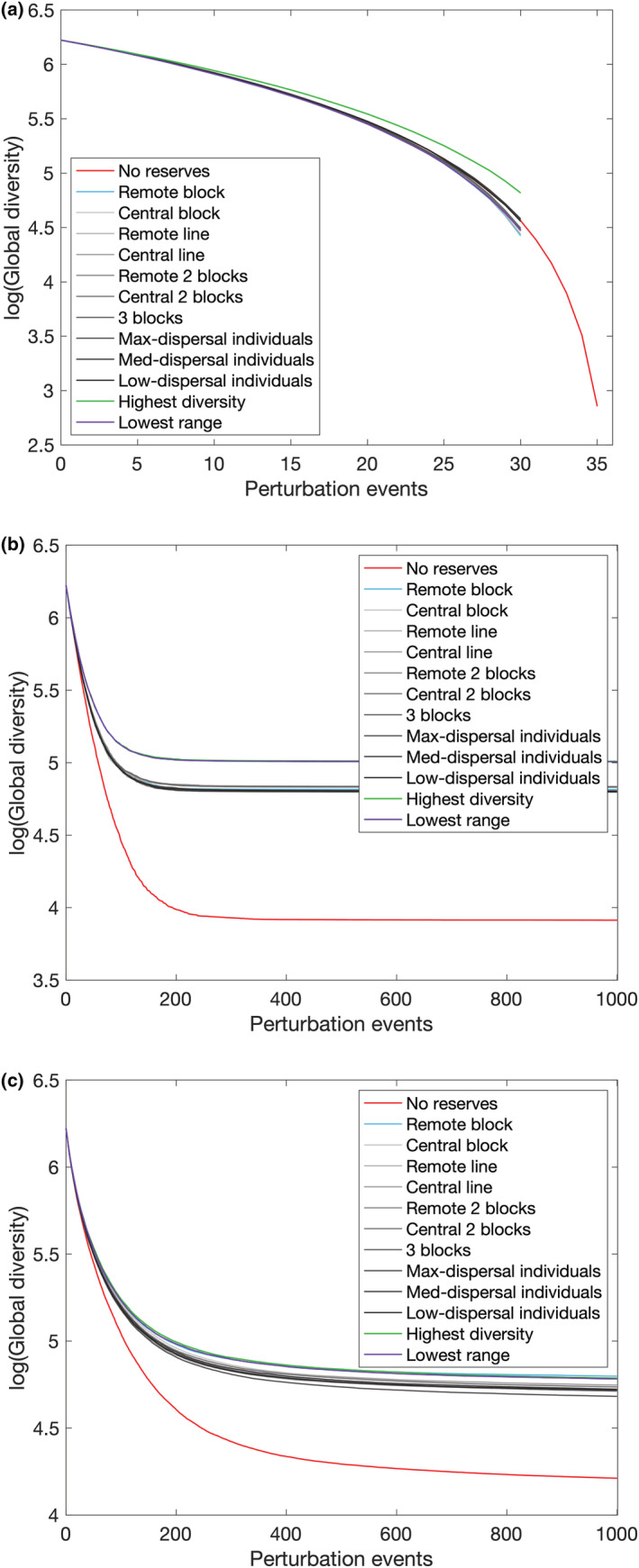
Selected average time series of perturbation sequences: (a) permanent random elimination, (b) temporary random elimination, (c) temporary random displacement.

Examining the time series of the perturbation sequences confirms that the final patterns are consistent across the course of the experiment. Protecting highest‐diversity patches is visibly beneficial by perturbation 10–15 in any case of permanent disruption (Figure 12a), while under temporary random elimination sequences, highest‐diversity and lowest‐range reserve choices are almost identical and superior to pre‐defined reserve placements by 30–40 perturbation events (Figure 12b). Temporary displacement demonstrates the benefits of these and remote block reserves by 150–200 perturbation events, which continue to be damaging right up to 1000 events (Figure 12c). Meanwhile, the meta‐communities approach equilibrium under elimination sequences by 150–300 events dependences on the reserve choice (Figure 12b).

## 8 CONCLUSION

A set of diverse trophic meta‐communities have been assembled on a spatial lattice using an eco‐evolutionary model, with the local ecosystems corroborating the community‐level network and structural patterns previously observed in similar models. These complex co‐evolved meta‐communities are utilized as the basis for perturbation experiments at both the species and community level, with all results applicable only to arrangements of low‐range species in spatially heterogeneous environments. In agreement with existing theory, the combined processes of dispersal and the resulting availability of multiple resources enhance the meta‐community's persistence against the artificial loss of any single species with relatively few secondary extinctions. However, invasion of the entire spatial network can be destructive (up to 12.8% of the global biodiversity) even if it is by a species that formerly existed within the meta‐community and it is re‐introduced to all patches with minimal population. In this model, large bodysize species demonstrated a greater probability of successful patch invasion. However, this advantage vanished if the same species were introduced to a meta‐network consisting only of the resources. In either case, co‐evolved species showed an advantage when re‐invading the meta‐communities over a species whose traits were randomly generated. However, this could be compensated for by increased bodysize when invading mature communities.

Habitat disruption due to human activity and development can be simulated by patch‐level disruption in a spatial meta‐network. Whether it is more damaging to displace the local populations of a patch than to eliminate them can be determined by measures of the ratio of at‐risk species within the patch and within neighboring patches where affected populations would be re‐settled. To reduce the ecological impact of anthropogenic habitat loss, optimal reserve placement is dependent on the mode of habitat disruption. Generally, reserves should dynamically target the highest‐diversity patches but if re‐colonization is possible then reserves that preserve the lowest average range species are also beneficial. If the populations of perturbed patches are displaced to neighboring patches, reserves should be situated in a remote region as a single large block to maximize the isolation of the interior from the invasion of displaced species. To investigate this effect further, future work would require a systematic study of reserves consisting of at least nine patches in larger spatial networks, so that the effect of isolating an interior patch in a 3×3 reserve block can be untangled from the effect of placing the reserve at the edge of accessible space. Other challenges remain to assemble model trophic meta‐communities of sufficient complexity, and in particular, work on eco‐evolutionary meta‐community models should employ a habitat mosaic with a small, fixed number of habitat types for the patches. This would facilitate a more realistic variable range of the species, allow for the modeling of real‐world geographies and testing the impact of habitat autocorrelation in space, systematic changes in habitat type, corridor placement, and for direct comparison with existing studies on thresholds of habitat loss (Pillai et al., 2011; Yin et al., 2017) for community collapse. We hope that future efforts will be able to address these and provide stronger recommendations for the principles of conservation of biodiversity and, in particular, the practical question of ideal nature reserve placement.

## AUTHOR CONTRIBUTION

**Gavin M. Abernethy:** Conceptualization (lead); data curation (lead); formal analysis (lead); investigation (lead); methodology (lead); project administration (lead); software (lead); visualization (lead); writing – original draft (lead); writing – review and editing (lead).

## ACKNOWLEDGEMENTS

The author would like to thank two anonymous reviewers for their useful and detailed comments that helped to revise the manuscript.

## FUNDING INFORMATION

Open access publication of this work was supported by the Wiley JISC agreement with the University of Stirling.

## Data Availability

The data that support the findings of this study are available at https://github.com/gavinabernethy/perturbation_responses, or from the corresponding author upon request.
